# Variant discovery in targeted resequencing using whole genome amplified DNA

**DOI:** 10.1186/1471-2164-14-468

**Published:** 2013-07-10

**Authors:** Amit R Indap, Regina Cole, Christina L Runge, Gabor T Marth, Michael Olivier

**Affiliations:** 1 Department of Biology, Boston College, Chestnut Hill, MA, USA; 2 Biotechnology and Bioengineering Center, Medical College of Wisconsin, Milwaukee, WI, USA; 3 Department of Otolaryngology and Communication Sciences, Medical College of Wisconsin, Milwaukee, WI, USA; 4 Texas Biomedical Research Intitute, San Antonio, Texas, USA

**Keywords:** Whole genome amplified DNA, Capture sequencing, Next generation sequencing, Variant discovery

## Abstract

**Background:**

Next generation sequencing and advances in genomic enrichment technologies have enabled the discovery of the full spectrum of variants from common to rare alleles in the human population. The application of such technologies can be limited by the amount of DNA available. Whole genome amplification (WGA) can overcome such limitations. Here we investigate applicability of using WGA by comparing SNP and INDEL variant calls from a single genomic/WGA sample pair from two capture separate experiments: a 50 Mbp whole exome capture and a custom capture array of 4 Mbp region on chr12.

**Results:**

Our results comparing variant calls derived from genomic and WGA DNA show that the majority of variant SNP and INDEL calls are common to both callsets, both at the site and genotype level and suggest that allele bias plays a minimal role when using WGA DNA in re-sequencing studies.

**Conclusions:**

Although the results of this study are based on a limited sample size, they suggest that using WGA DNA allows the discovery of the vast majority of variants, and achieves high concordance metrics, when comparing to genomic DNA calls.

## Background

There has been considerable focus in human genetics on characterizing rare variation in the human population, and the role these variants play in human diseases to account for the “missing heritability” in genome-wide association studies using common variants [1,2]. Until recently, the discovery of genetic variants was the rate-limiting step due to the prohibitive cost of sequencing large numbers of samples using traditional Sanger sequencing. Over the past five years, next generation sequencing (NGS) technologies have replaced traditional Sanger sequencing as the predominant method of DNA sequencing [3,4]. The main advantage of NGS over traditional Sanger sequencing is its cheaper cost and higher throughput. NGS has had a profound impact on the field of human genetics because it is now possible to sequence large numbers of individuals to fully describe the spectrum of human genetic variation, from common to rare variation [5]. In parallel to the developments of new sequencing technologies, improved methods have been developed to enrich specific subsets of the genome for next generation sequencing. While commonly referred to as exome sequencing, because in many cases protein coding regions have been enriched, in fact any portion of the genome can be chosen for target enrichment [6,7]. Capture sequencing allows many individuals to be sequenced for particular regions of interest, as opposed to whole genome sequencing a smaller number samples at the same cost [8]. This also provides greater sensitivity for SNP detection compared to whole genome sequencing [9]. Exome capture sequencing has yielded many successful examples for uncovering causative mutations in Mendelian disease [10,11], and describing the full extent of rare variation in protein-coding portions of the genome that whole genome sequencing may have missed because high-coverage, whole genome sequencing is still not common practice [12].

While the discovery of genetic variation is no longer a rate-limiting step for human genetic analysis, the application of NGS and sequence capture technologies can be limited by the amount of DNA available [13]. In particular, probands that have been collected for a clinical study maybe difficult to sample again. Previously collected DNA samples gradually decay in quality over time, and non-invasive collection techniques, such as buccal swabs, may result in insufficient amounts of DNA [13]. Several rounds of NGS or capture array sequencing may deplete original stock aliquots of samples. Whole genome amplification (WGA) is a method to overcome such challenges, and can yield micrograms of WGA DNA from nanogram starting amounts of template.

Previous studies have shown that WGA DNA performs well on high-density SNP genotyping arrays [14-16]. Three recent studies have investigated the use of WGA DNA in NGS. Murphy et. al. [17] investigated the use of a WGA protocol performed *in situ* on laser capture micro-dissection cancer cells for the discovery of structural variants in a tumor genome using Illumina mate-pair sequencing. Tao et. al. [18] showed that WGA DNA has favorable sequence capture metrics when comparing to genomic DNA when adapting the NimbleGen capture array for use on the Illumina GA sequencing platform. El Sharawy et. al. [19] investigated the use of WGA DNA in a NGS microdroplet-based PCR sample enrichment pipeline experiment of 384 exons with 3 HapMap samples. They showed there was strong genotype concordance with both genomic and WGA DNA SNP calls to HapMap III genotypes. In this paper we describe the results of variant calls using WGA DNA for a single sample for two separate capture sequencing experiments on the Agilent SureSelect platform, and compare them to variant calls made with genomic DNA for the same samples. While the results in this study are based on a limited number of samples, our results suggest that WGA samples have a high sensitivity in detecting variant alleles identified with genomic DNA, and can be used effectively in re-sequencing studies.

## Results and discussion

### Capture metrics of WGA and genomic DNA

We analyzed capture sequencing metrics of genomic and WGA sample pairs for two capture experiments, a chr12 custom array and a whole exome capture array. Additional file 1 contains capture metrics from the program CalculateHsMetrics from the software package Picard [20]. The average target coverage for the whole exome capture experiments were 92x (WGA) and 80x (genomic). The average target coverage for the chr12 capture experiments were 432x (WGA) and 224x (genomic). WGA samples in both capture experiments had a higher number of PF (passed filter) reads thus higher average target because they were sequenced in a separate flow-cell lane, while the genomic DNA samples were multiplexed. For both sequencing experiments a large percentage of reads were marked as duplicates, as the percentage of usable bases on target for each of the capture experiments does not exceed 40%. Despite the high duplicate read fraction both samples in the whole exome capture experiment had 80% targeted of bases with at least 20x coverage. For the smaller chr12 capture experiment, over 90% of targeted bases had at least 20x coverage.

Since the WGA capture experiments had a larger sequencing library compared to the genomic, a random subset of reads were selected from the starting fastq files to match the number of PF reads of the genomic sequencing library (see Additional file 1 and Methods). The average target coverage for the chr12 WGA subsetted BAM (342x) is higher than the chr12 genomic experiment, even though the starting number of PF reads is the same. This can be attributed to higher percentage of usable bases on target, as calculated with HsMetrics. Similarly, the whole-exome WGA subsetted BAM average target coverage (63x) is less than the genomic sample, despite starting with the same number of PF reads. The percent usable bases on target are lower in the whole-exome WGA subset than the whole exome genomic sequencing experiment.

**Figure 1:**
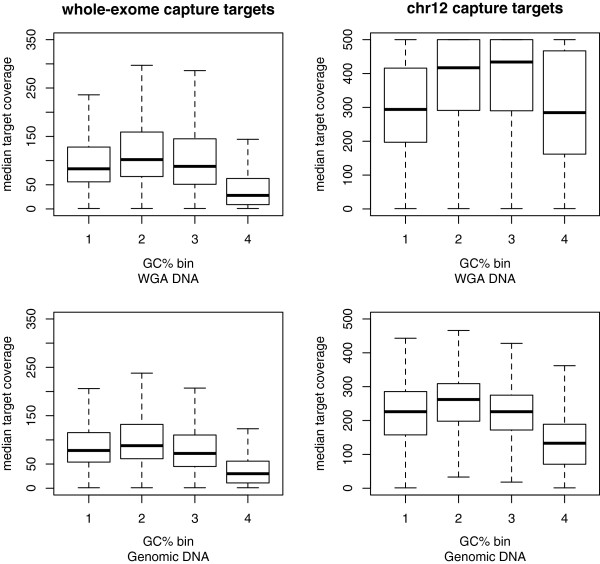
**Boxplots of median target coverage.** Boxplots of median coverage of targets binned according to quartiles of GC% of capture targets for chr12 and whole-exome capture experiments.

Next, we explored the relationship between GC% and median target coverage for both capture experiments. Previous studies have shown that lower sequencing coverage occurs in regions with high GC% [21]. GC% of targets for each capture experiment was calculated. Next, the targets were placed in four bins according to the first, median, and third quartiles of capture target GC%, based on the boxplots shown in Additional file 2: Figure S1. In addition to boxplots of GC% of capture targets of the two experiments, Additional file 2: Figure S1 shows the GC% of the whole genome and chr12 for comparison. Targets were placed in the appropriate bin and within each bin, a box plot of median target coverage was made for genomic and WGA DNA, as shown in Figure 1. The results show that for genomic DNA, chr12 capture targets in the fourth bin (with GC% greater 51%) have lower coverage than targets in the other three bins. For the corresponding WGA DNA, targets in the first (GC% less than 38%) and fourth bins have a similar distribution of median target coverage. Whole exome capture targets in the fourth bin (GC% greater 59%) had lower amounts of coverage than targets with lower GC% for both genomic and WGA samples. Since the chr12 capture targets were over a much smaller interval (3.87 Mbp), its harder to make any definitive statement regarding GC% and lower sequencing coverage, but the patterns of coverage seen in both capture experiments examined here are in line with previous studies [9,21].

### Overall variant counts and Venn analysis

Table 1 shows the counts and callset metrics of the individual SNP and INDEL callsets, after post-call filtering (described in the Methods). For all SNP callsets the dbSNP fraction is 98%. The overall transition-transversion (TsTv) ratio for the WGA and genomic chr12 callsets are 2.42 and 2.41 respectively. The overall TsTv ratio for the WGA and genomic whole-exome callsets are 2.83 and 2.82, respectively. The TsTv values of novel SNPs found in each of the capture experiments is considerably reduced, suggesting these may be false positive calls.

**Table 1:** Read coverages, SNP and INDEL summaries

**Dataset**	**chr12 WGA**	**chr12 Genomic**	**Whole-exome WGA**	**Whole-exome Genomic**
**SNPs**	4642	4592	29600	30316
**dbSNP %**	98.4	98.6	98.6	98.6
**TsTv overall**	2.42	2.41	2.83	2.82
**TsTv novel**	1.47	1.48	1.81	1.89
**TsTv known**	2.44	2.43	2.85	2.84
**INDELs**	491	482	2197	2215
**dbSNP %**	34.8	34.0	34.2	34.8

Read coverages, SNP and INDEL summaries.

**Figure 2:**
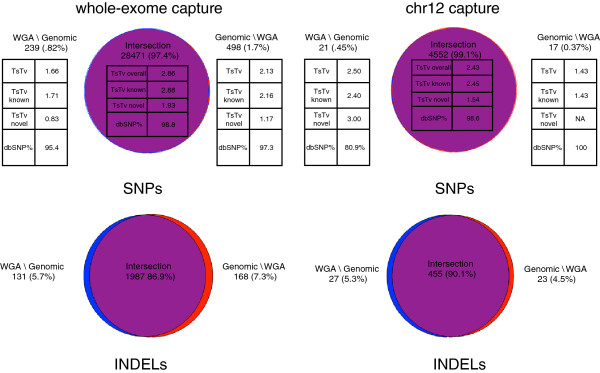
**Venn diagrams of SNP and INDEL variant calls.** Venn diagrams of SNP and INDEL variant calls. The top row also shows TsTv ratios and dbSNP fractions of SNPs in each portion of Venn diagram. The notation A\B means elements in callset A but not in callset B(i.e. unique to fraction A).

We performed Venn analysis of the WGA and genomic callsets to see how variants overlapped based on coordinate intersection. Figure 2 shows four Venn diagrams for SNP and INDEL sites in each of the capture experiments. Visual inspection indicates there is a high fraction of site-level concordance of SNP calls, with 97% and 99% of the union of SNP sites lying in the intersection for the whole exome and chr12 capture callsets. Slightly lower numbers of 87% and 90% were found for INDEL sites. Overall TsTv ratios for SNPs in the intersection were similar to those calculated for each individual callset. TsTv ratios of novel sites were slightly higher in the intersection, when compared to the original callsets. The TsTv values of the genomic and WGA unique fractions for the whole-exome capture experiment are considerably lower, suggesting these are lower quality calls. The unique fractions of the chr12 capture experiment are much smaller, making it difficult to interpret the differences in value of their TsTv ratios.

### Downsampling alignments and subsetting reads

Since the WGA samples were run as a single lane but the genomic samples were multiplexed, we downsampled reads from each BAM to examine the effect of coverage on the numbers of discovered variants. A total of 100 bootstrap sub-samples of reads were performed (see Methods). In addition to downsampling the reads from the aligned BAM file, a subset of fastq reads were chosen at random to match the starting number PF reads in the genomic library for both experiments (see Additional file 1).

**Figure 3:**
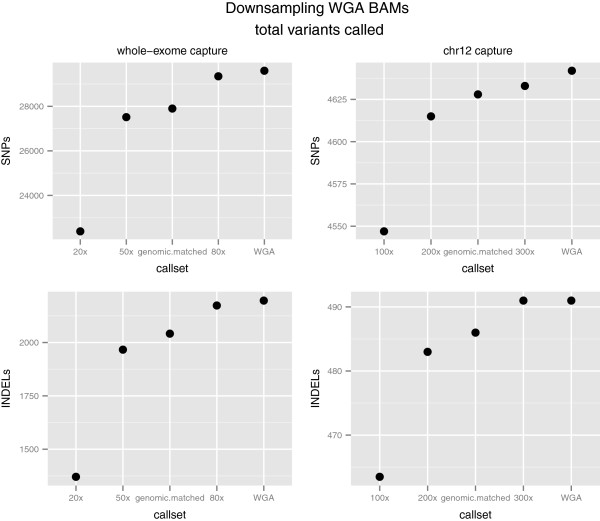
**Number of variants discovered in downsampled and subsetted WGA BAMs.** Median number of SNPs and INDELs called from 100 bootstrap subsampled BAM files from whole-exome and chr12 capture experiments for WGA DNA samples. Plot also includes number of variants discovered in the WGA subsetted BAM that matched the starting read count of the genomic sample.

Figure 3 shows the median number of variants discovered as a function of average target coverage for SNPs and INDELs, for each capture experiment. The randomly chosen subset of WGA reads to match the number of PF reads in the genomic sequencing experiment is shown as genomic.matched on the x-axis, and sorted in ascending order of target coverage. As expected, downsampling BAMs reduces the number of called variants, with the original WGA BAM having the largest number of called variants. The datapoint that most closely matches the target coverage of the non-WGA sample is 80x for the whole-exome plot. The median number of SNPs and INDELs found (29350 and 2174) closely match the numbers of variants found the in genomic derived variant calls listed in Table 1. The datapoint that most closely matches the target coverage non-WGA sample is 200x for the chr12 plot. The median number of SNPs and INDELs found (4615,483), again closely match what was found in the genomic derived calls listed in Table 1.

### Genotype concordance

We used two measures of genotype concordance, non-reference sensitivity (NRS) and non-reference discrepancy (NRD) [22,23], shown in Additional file 3: Figure S2, to compare genotypes made with WGA and genomic DNA. NRS measures the proportion of sites called variant in the comparison callset (genomic) that are also called variant in the evaluation callset (WGA). NRD measures the proportion of differing genotypes between the WGA and genomic callsets, at sites called in both data sets, excluding concordant homozygous reference calls.

The NRS and NRD values for SNPs and INDELs for each capture experiment are shown in Table 2 and the concordance matrices from which they were calculated are shown in Additional file 4: Figure S3. For the chr12 capture experiment, of the 17 sites that contribute to the decrease in SNP NRS of the WGA call set, six are heterozygous sites in the genomic DNA that were not called in WGA DNA. Of the 28 sites contributing to the decrease in INDEL NRS, 18 were heterozygous genotypes in genomic DNA, that were evenly split as homozygous reference or no calls in WGA DNA. For the 13 sites contributing SNP NRD, eight were WGA heterozygous sites, called homozygous non-reference in genomic DNA. The greatest contribution to INDEL NRD came from sites that were called heterozygous in WGA DNA, but homozygous reference in genomic DNA.

**Table 2:** Genotype concordance metrics

**Dataset**	**NRS**	**NRD**
whole-exome capture SNPs	98.28	0.63
whole-exome capture INDELs	91.17	13.46
chr12 capture SNPs	99.63	0.29
chr12 capture INDELs	94.07	10.7

NRS and NRD values for WGA derived whole-exome and chr12 capture call sets when comparing to genomic call sets.

**Figure 4:**
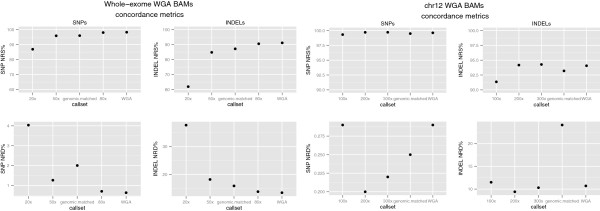
**Genotype concordance metrics of downsampled subsetted WGA BAMs.** Median values of NRS and NRD metrics for SNP and INDEL variants calculated from 100 bootstrap subsampled BAM files from whole-exome and chr12 capture experiments for WGA DNA samples. Plot also includes NRS and NRD metrics of the WGA subsetted BAM that matched the starting read count of the genomic sample.

Next, genotype concordance for each bootstrap downsampled chr12 capture BAM was calculated by comparing its calls to the ones made from the original genomic BAM file. NRS and NRD values were summarized by calculating their median value across all 100 downsampled BAMs. In addition, NRS and NRD of the subsetted WGA BAM was calculated by comparing its genotypes to the original genomic BAM. Figure 4 shows the affect of downsampling and subsetting on genotype concordance metrics. Unexpectedly, two of the three downsampled datasets have slightly higher SNP and INDEL NRS values than the original WGA callset. This includes the NRS of the 200x downsampled BAM, which most closely matches the coverage of the genomic sample. Similarly, the original WGA callset has a higher NRD values than some of the lower coverage, downsampled BAMs (including the 200x downsample BAM). The INDEL NRD for the genomic matched WGA BAM is clearly an outlier on the graph. This might be attributed to sampling error, but since the WGA fastq files were subsetted only once, its difficult to say. This unexpected pattern can potentially be attributed to the smaller capture interval in the chr12 experiment and the fewer numbers of variants called, as the relationship between concordance metrics and lower coverage, downsampled BAMs is clearer in the whole-exome capture experiment (see below). Also, since a technical replicate of genomic sequencing was not performed, it’s difficult to ascertain what the expected genotype discrepancies should be between genomic and WGA derived variant calls.

The NRS and NRD values and the genotype concordance matrix from which they were calculated for the whole-exome capture experiment are also shown in Table 2 and Additional file 4: Figure S3, respectively. Of the 498 sites that contribute to the decrease of SNP NRS of the WGA call set, the majority come from sites either called heterozygous or homozygous non-reference in genomic DNA but were no calls in WGA DNA. The majority of sites contributing to the decrease of INDEL NRS come from sites called heterozygous in genomic DNA, but called homozygous reference in WGA DNA. Sites contributing most to SNP NRD are heterozygous calls in genomic DNA, called homozygous reference in WGA DNA, for both SNP and INDEL variants. The concordance metrics of the WGA whole exome downsampled BAMs to original genomic DNA calls, also shown in Figure 4, reinforce the intuitive expectation that the lower coverage WGA callsets result in higher NRD and lower NRS values. The one exception is the SNP NRS of the genomic matched subsetted WGA BAM, which had a NRD value of 2%. This could be attributed to sampling error, since the subsetting was only performed once, and not multiple times like the downsampling. The SNP and INDEL NRS of the downsampled 80x BAMs, which match the average coverage of the genomic BAM, are only slightly lower than the original WGA BAM. Also, the SNP and INDEL NRD values are slightly higher than the original WGA BAM. Still, in each comparison, the original WGA call set had the lowest NRD and highest NRS values relative to lower coverage downsampled and subsetted callsets. As with the chr12 experiment, the genomic sequencing was not repeated, so it difficult to quantify the expected genotype discrepancies and sensitivity of the WGA derived variant calls.

**Figure 5:**
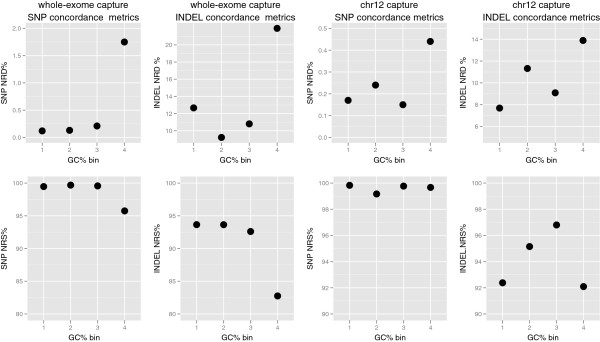
**Genotype concordance metrics as a function of GC%.** NRS and NRD of variants binned according to GC% based on quartiles of GC% in capture targets. First row shows NRD values for SNP and INDELs and second row shows NRS values of SNPs and INDELs for each of the chr12 and whole-exome capture experiments.

Targets with higher amounts of GC% have lower amounts of median target coverage for both capture experiments and both types of DNA, as described above. Figure 5 shows NRS and NRD metrics for each bin, based on GC% of targets. For the original WGA whole-exome callset, the greatest number of genotype discrepancies and lowest detection sensitivities, for both SNP and INDEL variants, occur in targets with the highest GC%. The patterns are less clear for the original WGA chr12 callset, again most likely attributable to the smaller size of capture region. For both SNP and INDEL variants, the greatest numbers of genotype discrepancies are in targets with the highest GC%. The pattern is less clear for variant detection sensitivity, INDELs in target regions with the highest amount of GC% have the lowest sensitivity, but this is not true for SNPs.

### Allele bias in SNP variant calls

**Figure 6:**
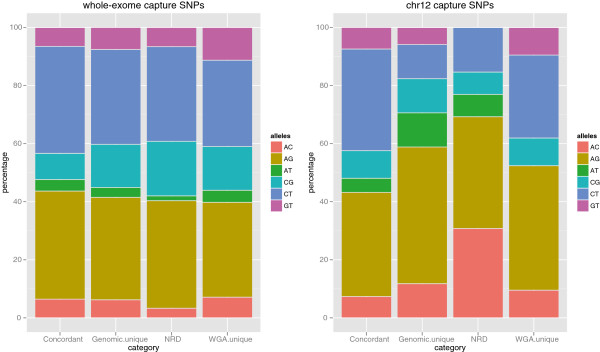
**Allelic proportions of SNPs.** Allelic proportions of whole-exome and chr12 capture SNPs in each of four categories: concordant genotypes, unique sites in original genomic and WGA call sets, and discordant genotypes contributing to NRD for chr12 and whole-exome capture experiments when comparing WGA derived SNP genotypes to genomic DNA callset.

To investigate whether there is any evidence of allele bias in SNP variant calls, all calls from the original chr12 and whole-exome WGA datasets were divided into four groups: concordant genotypes, unique genomic calls (these are sites that contribute to NRS), discordant genotypes (these are sites that contribute to NRD), and WGA unique. In each group, the percentage of each six possible reference/alternate allele combinations was calculated. The results are shown in Figure 6. We tested to see if there were statistically different proportions of each reference/alternate allele combinations (see Methods) between the four groups. The resulting p-values are in Additional file 5. For the whole-exome capture SNPs, there was a significant difference in proportion between concordant CG SNPs and each of the three other categories. Also, there was a significant difference in proportion of GT SNPs between concordant and WGA unique categories. For the chr12 capture set there was no significant difference in proportion of SNPs between any of the four categories for each of the 6 different allele combinations. The interpretation of the statistical analysis of allele bias must be tempered by the fact that the analysis is based on a small sample size of matched genomic / WGA samples, lack of technical replicates, and the reduced target region for the chr12 capture experiment. But even with this in mind, results suggest that allele bias does not play a significant role in SNP variant discovery with WGA DNA.

### Validation of SNP variant calls

Sequencing derived SNP variant calls were validated by comparing genotypes to Affymetrix 6.0 Human SNP array genotypes for the same sample. The 6.0 array has over 900,000 variants covering the whole genome, hence only those array genotypes that overlapped a capture target interval were examined. For the whole-exome capture array there were a total of 11831 overlapping SNPs and for the custom chr12 capture array there were a total of 1435 overlapping SNPs. See the Methods section for more details. The NRS and NRD metrics of the WGA and genomic sequencing based SNP genotypes when compared to Affymetrix 6.0 SNP array genotypes for both capture experiments is shown in Table 3. The NRD for the WGA whole-exome capture sequencing derived genotypes when compared to the SNP array genotypes is 1.3% and the NRS value is 97.78%. The NRD for the genomic whole-exome capture sequencing derived genotypes when compared to the SNP array genotypes is 1.6% and the NRS value is 97.66%.

**Table 3:** Affymetrix SNP array genotype concordance metrics

**Dataset**	**NRS**	**NRD**
WGA whole-exome capture SNPs	97.78	1.30
Genomic whole-exome capture SNPs	97.66	1.60
WGA chr12 capture SNPs	82.60	22.20
Genomic chr12 capture SNPs	83.00	22.60

NRS and NRD values for whole-exome and chr12 capture call sets when comparing to Affymetrix 6.0 genotypes.

The concordance matrix for the WGA whole-exome comparison to capture array genotypes is shown in the top panel and the genomic concordance matrix is shown in the bottom panel in Additional file 6: Figure S5. For sites that contribute to a decrease in whole-exome capture NRS, the read coverage and pileup of bases was investigated. For the 94 sites in the WGA whole-exome capture call set that contribute to a decrease in NRS, 20 had minimal coverage and were called homozygous reference. The remaining sites have an overwhelming majority reads with mapping quality 0 spanning the SNP position and were not called. Similarly, for the 100 sites that contribute to the decrease in NRS in the genomic DNA whole-exome capture derived genotypes, 29 had minimal coverage and were called homozygous reference. The remaining sites had reads spanning the SNP position with mapping quality values of zero and not called. There are a total of 68 SNP positions common to both WGA and genomic callsets that contribute to a loss of NRS when comparing the Affymetrix SNP array genotypes.

The NRS and NRD values when comparing the WGA, chr12 capture sequencing SNP genotypes to the SNP array genotypes are 82.6% and 22.3%. The NRS for the genomic DNA, chr12 capture SNP genotypes when compared to SNP array genotypes is 83% and the NRD is 22.6%. The concordance metrics for the chr12 custom array SNP genotypes when compared to the SNP array derived genotypes is shown in Additional file 7: Figure S6. The top panel shows the concordance matrix for the WGA chr12 capture array and in the bottom panel is the genomic chr12 concordance matrix. For both comparisons, the majority of sites that contribute to the loss of sensitivity in the sequencing derived SNP calls are sites that were called heterozygote on the genotyping array. Careful visual inspection and examination of read pileups in the WGA and genomic BAM files revealed no evidence of an alternate allele and hence were called homozygous reference. There are total of 144 SNP position common to both WGA and genomic call sets that contribute to a loss NRS when comparing to the Affymetrix SNP array genotypes.

### Allele bias in SNP variant validation calls

**Figure 7:**
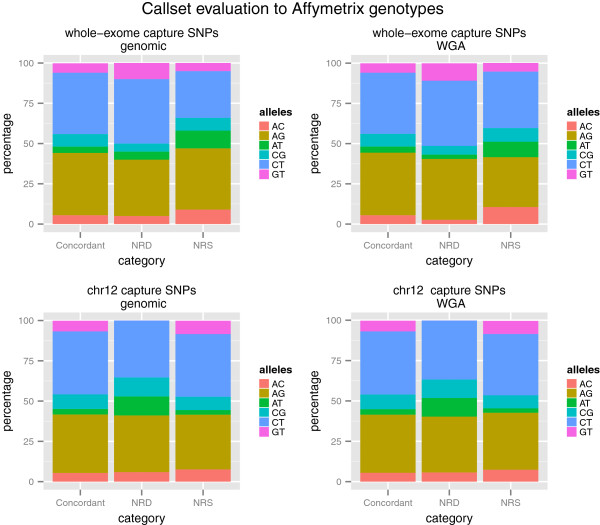
**Allelic proportions of Affymetrix SNPs.** Allelic proportions of whole-exome and chr12 capture SNPs in each of 3 categories: concordant genotypes, NRD contributing, and NRS contributing for chr12 and whole-exome capture experiments when comparing WGA and genomic SNP call sets to Affymetrix SNP array genotypes.

To investigate if there were any biases in the comparisons of the sequencing derived genotypes to the Affymetrix array based genotypes the percentage of each six possible reference/alternate allele combinations was calculated in sites that contributed to concordant, NRS, and NRD categories. The results are shown in Figure 7. To test if there were statistically different proportions of each reference/alternate allele combinations between groups we applied the same pairwise.fisher.test when comparing the WGA derived SNP calls to the genomic derived SNP calls (see Methods). The resulting p-values of the analysis are in Additional file 5. The only significant differences in proportion detected were AT SNPs when comparing the chr12 genomic and whole exome capture calls to the corresponding Affymetrix array derived genotypes.

## Conclusions

The study described here provides an in-depth assessment of the suitability of WGA DNA for targeted resequencing and variant discovery using next generation sequencing. We evaluated whole exome as well as targeted genomic enrichment using Agilent SureSelect technology, and compared findings from WGA samples to results obtained with genomic DNA from the same individual, as well as validated a subset of SNP variant calls with Affymetrix SNP array genotypes. Overall, Venn analysis showed that the numbers of SNPs and INDELs called in the whole exome and chr12 capture callsets using WGA or genomic DNA is very similar, with the vast majority of variant sites shared between datasets. The concordance metric NRS demonstrates that using WGA DNA has high sensitivity for SNP sites with values of 98.28% and 99.63% for the whole exome and chr12 sequence capture callsets, respectively. The NRS for INDELs is lower at 91.17% and 94.07%. SNP NRD values for the whole-exome and chr12 callset were both less than 1%, but were an order of magnitude higher for INDEL calls. The lower values of these metrics may be due to slight differences in alignment of reads between genomic and WGA DNA in regions that contain INDEL variants. The majority of discrepant genotypes between WGA and genomic DNA involve heterozygous genotypes and statistical analysis suggests that these are enriched for GC alleles, at least in the whole-exome capture data. Validating a subset of the SNP made with genomic and WGA DNA that overlap sites on the Affymetrix 6.0 SNP array showed high sensitivity and high genotype accuracy for the whole exome capture callset. The sensitivity and genotype concordance numbers for the chr12 capture array were not as high, but the loss of sensitivity can be explained by lack of evidence of the alternate allele in the read pileup or poor zero mapping quality values spanning the SNP position. Downsampling and subsetting of reads to achieve lower coverage in WGA callsets (or match the starting number reads in the genomic sequencing experiment) consistently resulted in lower genotype concordance and sensitivity metrics for the whole exome capture experiment, in contrast to the chr12 capture experiment. This difference may be due to statistical fluctuations of read sampling in the downsampling process, combined with the much smaller size of the chr12 capture region. Coverage and concordance metrics correlated with GC% of target intervals, with target intervals above the 3rd quartile of each respective capture array having less coverage and poorer concordance metrics. Our work complements the study of ElSharawy [19] who used a greater number of matched genomic / WGA samples in showing both genomic and WGA samples had high concordance and sensitivity metrics to HapMap III sites, but whose study examined only 384 exons. A limitation of our study is that we only have 1 genomic/WGA sample pair for each of the capture experiments, and the chr12 experiment captured a much smaller region of genomic DNA. Since the genomic sequencing was not repeated, we cannot know the expected discrepancy for a technical replicate, but we were able to validate a subset of our SNP calls that overlapped sites on the Affymetrix SNP array. Thus, our conclusions about allele bias, and the relationship between GC% content and genotype concordance must be taken with caution, but overall suggest that WGA samples can be used effectively in re-sequencing studies and thus offer a promising alternative for variant discovery studies using archived DNA.

## Methods

### WGA and genomic DNA sample preparation

Two sample sets were analyzed in this study. One sample was from a family cohort [24] that was sequenced for a 3.87 Mbp region on chr12 using a custom designed SureSelect capture array from Agilent. The second sample was from a single family that was whole exome sequenced using the Agilent SureSelect All Exon kit. In both cases, the genomic DNA was originally isolated from blood samples. A REPLI-g Mini Kit (Qiagen) was used to prepare WGA DNA from 15 ng of starting genomic DNA.

### Sequence capture

We used two different Agilent SureSelect kits to perform sequence capture on the samples used in this study. The first was a custom array designed to capture a 3.87 Mbp region on chromosome 12. The second was an Agilent SureSelect All Exon kit designed to capture at total of 49.4 Mbp of exonic sequence spanning the whole genome. The standard Agilent SureSelect protocol for Illumina paired-end sequencing was used which requires 3 of micrograms of starting genomic DNA.

### DNA sequencing

Samples were paired-end sequenced on an Illumina GAII machine with read lengths of 101 bp. with insert size for the genomic and WGA whole exome capture samples being each 370 bp, respectively. Insert sizes for the genomic and WGA chr12 capture samples were both 320 bp, respectively. Both sets of genomic DNA samples were multiplexed with other samples not part of this study, while each of the corresponding WGA DNA samples were sequenced in an individual flow cell lane. Fastq files were generated via the Illumina CASAVA pipeline v1.8. The starting number of passed filter reads is shown in Additional file 1, as well as additional metrics of capture experiments.

### Bioinformatics pipeline

We applied the same bioinformatics pipeline to WGA and genomic DNA samples as shown in Additional file 8: Figure S4. All programs from the Genome Analysis Toolkit (GATK) were from version v1.6-5-g557da77 [22]. All programs from Picard were from v1.50 [20]. Fastq files were aligned to the human reference sequence GRCh37 with the program MOSAIK v2.0.113q [25]. Parameter values to MosaikAligner were as follows: -act 35, -bw 37, -mhp 200 -mm 14. Capture metrics for the whole exome and chr12 capture experiments were calculated using the program CalculateHsMetrics in Picard. Base quality scores were recalibrated with the GATK programs CountCovariates and TableRecalibration. PCR duplicates were marked using the program MarkDuplicates, which is part of Picard. SNP and INDEL variants were discovered using the GATK program UnifiedGenotyper. Parameters used for running UnifiedGenotyper were as follows: -stand_call_conf: 10, -stand_emit_conf: 30, -glm: BOTH, -out_mode: BOTH, -hets:.001. Each member of the WGA/genomic sample pair was called independently as a single sample. UnifiedGenotyper was given the target capture intervals of each experiment and only made variant calls in these intervals when calling INDEL and SNP variants. SNP variant calls were filtered using the GATK program VariantFiltration with the following filtering parameters:

$$\begin{array}{c} ((\text{MQ}0/(1.0^{*}\text{DP}))>0.05)||\text{DP}<5||\text{QUAL}<30.0||\text{QD}\\ \;<5.0||\text{HRun}>5.0||\text{SB}\geq -0.10 \end{array}$$

INDEL variant calls were filtered with the following:

$$((\text{MQ}0/(1.0^{*}\text{DP}))>0.05)||\text{SB}\geq -1.0||\text{QUAL}<10$$

Where MQ0 = Number of reads with mapping quality zero, DP = depth of coverage, QUAL = Phred scaled quality score, HRun = Largest contiguous homopolymer run of variant allele in either direction, QD = Variant Confidence/Quality by Depth, and SB = Strand Bias.

### Downsampling and subsetting of reads in WGA and genomic BAM files

To investigate the relationship between sequence coverage and number of variants discovered, aligned reads from both WGA BAM files were downsampled to different levels average target coverage using the Picard v1.50 program DownsampleSam. Since UnifedGenotyper restricted its variant calling to target capture interval regions, only aligned reads that had a minimum 1-bp overlap with a target interval were considered in the downsampling process by removing off target alignments by using the pairToBed program in BEDTools package [26] For the chr12 WGA BAM, 100 downsampled BAM files were generated with average target coverages of 100x, 200x and 300x, respectively. For the whole-exome WGA BAM, 100 downsampled BAM files were generated at coverage levels of 20x, 50x, and 80x. Since the WGA prepared samples had higher sequence coverages, the coverage range of the downsampled BAMs were chosen so they would closely overlap the coverage of the original genomic DNA sample. Due to the stochastic nature of the downsampling process, as well as variation in capture efficiency between targets, it was difficult to get exact match in the number of reads between WGA and genomic BAMs. The number of reads needed to achieve a desired coverage was determined by solving this equation: C =(N × L)/G, where C is the coverage, N is the number of reads, G is the size of the genome (in this case the total length in base pairs of capture array targets), and L is the read length value (101 bp).

In addition to downsampling the reads from the WGA BAMs for both capture experiments, an exact number of read pairs were randomly sampled from the initial WGA fastq files to match the starting number of genomic DNA fastq read pairs. This was accomplished by writing a Python script that randomly selects a specified number of read pairs from a fastq file. Once the subset of fastq read pairs were selected they were put through the same bioinformatics pipeline applied to the original data.

### Callset comparison metrics

We compared the variant calls from genomic and WGA using three types of metrics. The first was site level intersection to see if the same genomic position was called variant in both callsets. The other two types of metrics were non-reference sensitivity (NRS), and non-reference discrepancy (NRD), shown in Additional file 3: Figure S2. NRS measures the fraction of sites called variant in the comparison callset that are also called variant in the evaluation callset. For this study the evaluation callset are the WGA variant calls and the comparison callset are the genomic variant calls. Sites called homozygous reference or no-call in the evaluation calls, but were variant in the comparison callset reduce NRS. NRD measures the accuracy assigned genotypes called by both datasets. It excludes concordant homozygous reference calls. To calculate these values, the VCF files of the WGA and genomic callsets were merged using the GATK program CombineVariants and then calculated in Python. Only sites that have PASS in the filter column of the individual VCFs are evaluated when calculating NRS and NRD from the CombineVariants derived VCF.

### SNP validation with Affymetrix 6.0 Human SNP array

The SNP variant calls for WGA and genomic DNA for both capture sequencing experiments were compared to Affymetrix 6.0 Human SNP array derived genotypes for the same samples. SNP array genotypes were called with Birdseed v2. The 6.0 Human SNP array contains a genomewide collection of more than 900,000 sites. For a SNP array variant to be included in the validation analysis it must overlap a target region on the capture array and have a confidence score of at least 0.05. Only those variants that met these two conditions were considered. Based on these criteria there were a total of 11831 SNPs on the 6.0 array that overlapped the whole exome capture targets and 1435 SNPs that overlapped the custom chr12 capture targets. Similar to the comparison of WGA calls to genomic DNA calls, the VCFs of sequencing and array derived genotypes were merged using the GATK program CombineVariants. The sequencing derived genotypes were evaluated by comparing them to the array based genotypes and the NRS and NRD concordance metrics were calculated. Only sites that have PASS in the filter column of the individual VCFs were included when calculating NRS and NRD from the CombineVariants derived VCF.

### Statistical analysis of allele bias in SNP calls

For both the whole-exome and chr12 capture experiments, genomic and WGA SNP call sets were merged, and then placed into 4 categories: concordant, uniquely called genomic, differing genotypes (NRD contributing), and WGA uniquely called SNPs. The counts of each of the 6 possible allele combinations in each category were tallied. To test the null hypothesis that the proportion of SNPs are equal across all 4 categories, the pairwise.fisher.test using the Bonferroni correction method was applied in succession to each of the 6 possible allele combinations in R [27]. The pairwise.fisher.test is part of the CRAN R package fmsb [28]. The significance level *α*=.05 was chosen. Additional file 5 contains an Excel sheet with the of p-values for the whole-exome and chr12 capture experiments.

A similar analysis was performed when comparing the sequencing derived SNP calls to Affymetrix array derived genotypes for genomic and WGA capture experiments. The sequencing and Affy callsets were merged (only SNPs on the Affymetrix array that overlapped a target capture region were included) and placed into concordant, NRS, or NRD contributing categories. Additional file 5 has the table of p-values for the whole exome and chr12 comparisons to the array based genotypes.

### Sample ascertainment

All samples and protocols for this study have been reviewed and approved by the IRB of the Medical College of Wisconsin. In accordance with the approved protocols, all participants provided written informed consent to participate in the study. Only adult individuals were included in the study.

## Abbreviations

WGA: Whole genome amplification; NRS: Non-reference sensitivity.

## Competing interests

Authors declare no competing interests.

## Authors’ contributions

MO conceived the experiment, RC generated the data, CLR provided DNA samples and financial resources, ARI analyzed the data and wrote the paper with edits from MO and GTM. All authors read and approved the final manuscript.

## Supplementary Material

Additional file 1 **Excel table containing information about each of the capture experiments.** Excel table containing information about each of the capture experiments as summarized from Picard CalculateHsMetrics. Click here for file

Additional file 2 **Figure S1.** Boxplot of GC%. Boxplots summarizing GC% of whole-exome and chr12 capture targets as well as overall GC% of the whole genome and chr12 for comparison. For whole genome and whole chromosome 12, GC% was calculated in 10 kbp windows with a 5 kbp overlap. Click here for file

Additional file 3 **Figure S2.** Calculating NRS and NRD genotype concordance. Figure shows how concordance metrics of non-reference discrepancy (NRD) and non-reference sensitivity (NRS) are calculated. Click here for file

Additional file 4 **Figure S3.** Genotype concordance matrices. Figure shows genotype concordance matrices for chr12 and and whole-exome SNP and INDEL callsets from which concordance metrics of NRS and NRD were calculated from. Click here for file

Additional file 5 **Excel file with results of allele bias statistical analysis.** Excel worksheet containing the computed p-values for the allele bias statistical analysis. Click here for file

Additional file 6 **Figure S5.** Affymetrix genotype concordance matrices whole exome. Genotype concordance matrices of WGA and genomic DNA SNP calls to Affymetrix genotypes for the whole exome capture experiment. Click here for file

Additional file 7 **Figure S6.** Affymetrix genotype concordance matrices chr12. Genotype concordance matrices of WGA and genomic DNA SNP calls to Affymetrix genotypes for the chr12 capture experiment. Click here for file

Additional file 8 **Figure S4.** Bioinformatics pipeline. The bioinformatics pipeline applied to each of the genomic and WGA DNA samples for each of the capture experiments analyzed in this study. Click here for file
