# Supplementary figures and images for: Variant discovery in targeted resequencing using whole genome amplified DNA
Source: BMC Genomics. 2013 Jul 10;14:468. doi: 10.1186/1471-2164-14-468 (PMC3716764; doi:10.1186/1471-2164-14-468)

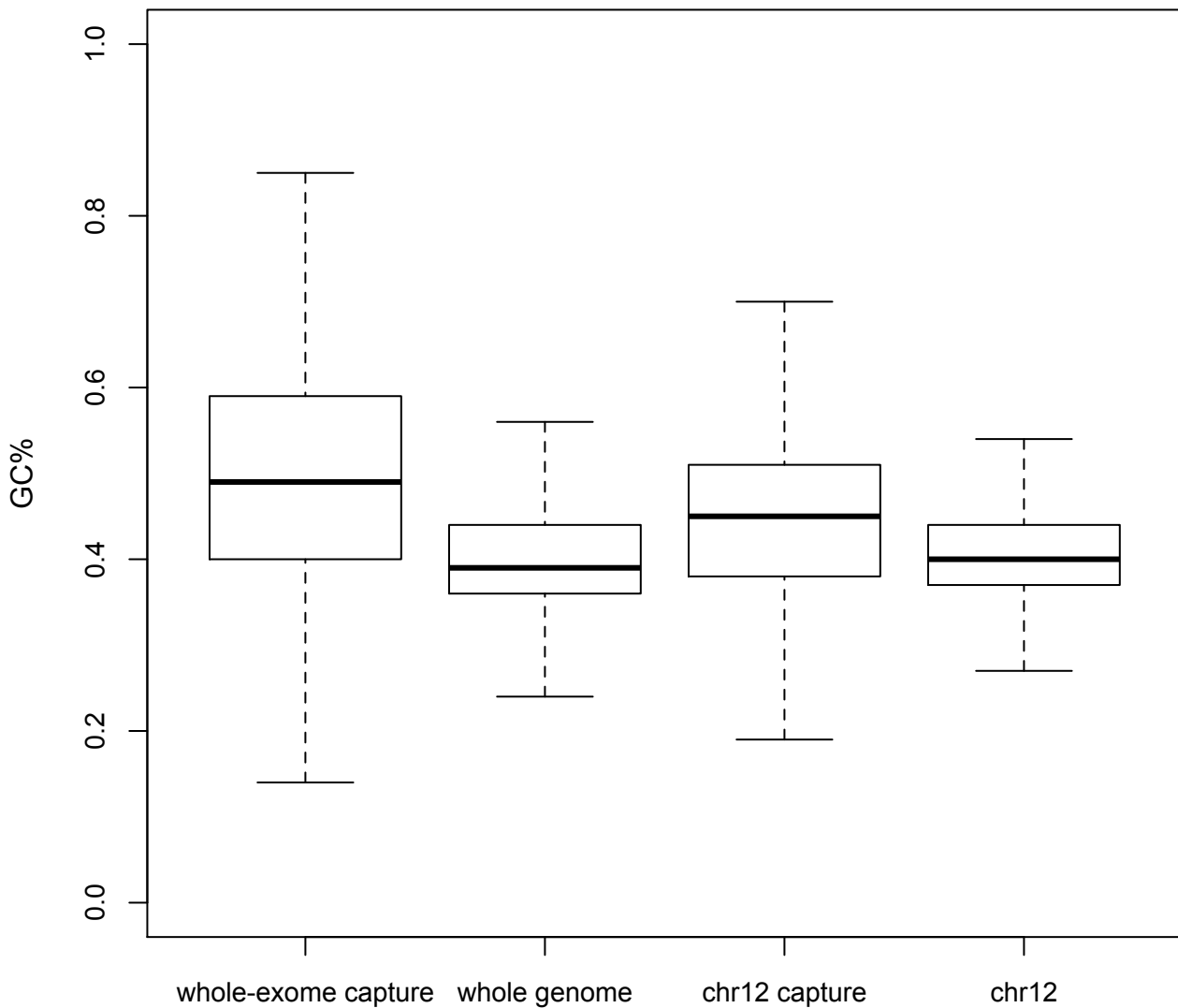

Supplement: Additional file 2 — Figure S1. Boxplot of GC%. Boxplots summarizing GC% of whole-exome and chr12 capture targets as well as overall GC% of the whole genome and chr12 for comparison. For whole genome and whole chromosome 12, GC% was calculated in 10 kbp windows with a 5 kbp overlap. [file 1471-2164-14-468-S2.pdf]

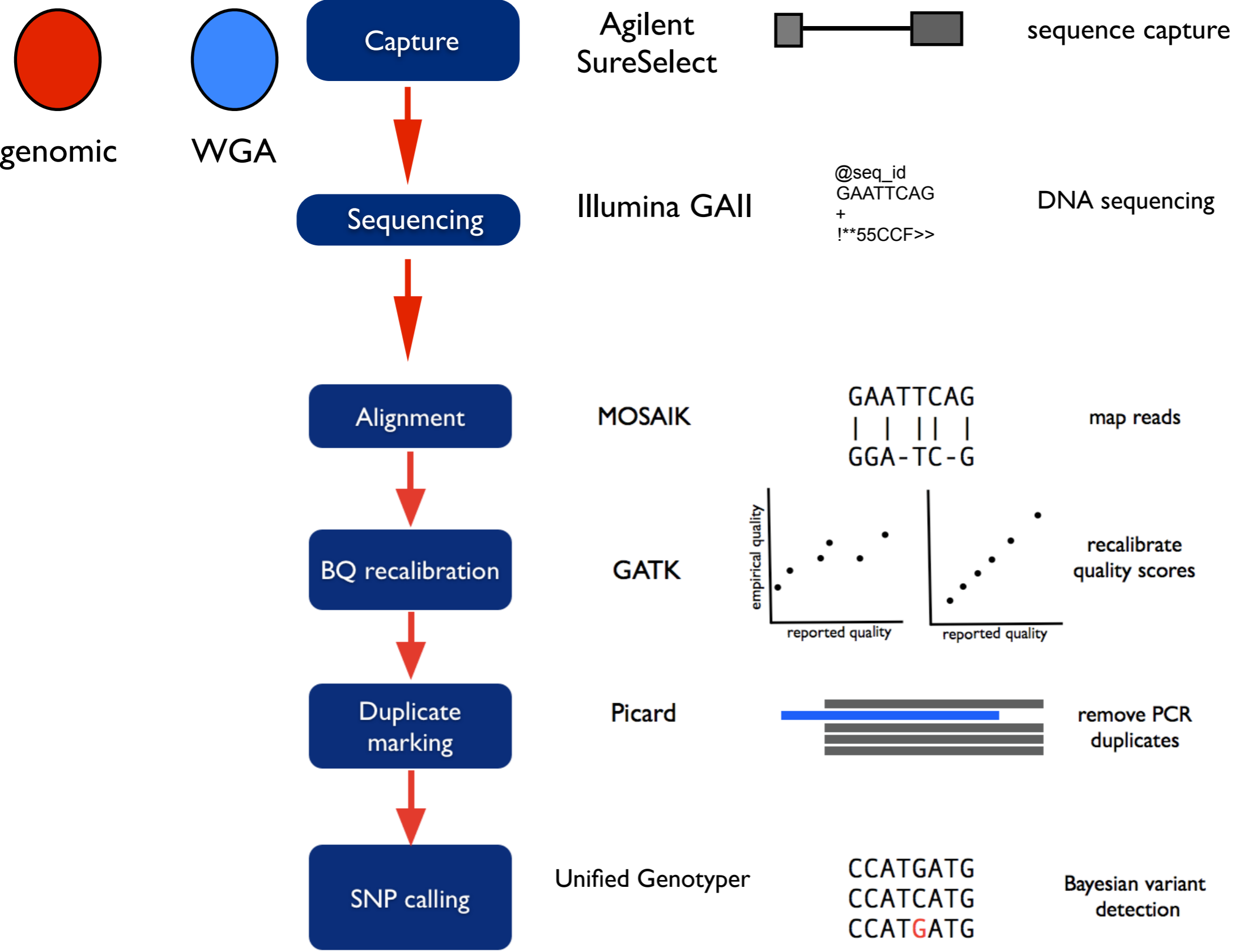

Supplement: Additional file 8 — Figure S4. Bioinformatics pipeline. The bioinformatics pipeline applied to each of the genomic and WGA DNA samples for each of the capture experiments analyzed in this study. [file 1471-2164-14-468-S8.pdf]
