# Supplementary material for: Variant discovery in targeted resequencing using whole genome amplified DNA
Source: BMC Genomics. 2013 Jul 10;14:468. doi: 10.1186/1471-2164-14-468 (PMC3716764; doi:10.1186/1471-2164-14-468)
Supplement: Additional file 3 — Figure S2. Calculating NRS and NRD genotype concordance. Figure shows how concordance metrics of non-reference discrepancy (NRD) and non-reference sensitivity (NRS) are calculated. [file 1471-2164-14-468-S3.pdf]

WGA variant calls (evaluation)

genomic variant calls (comparison)

	AA	AB	BB	nocall
AA		1	2	
AB		3	4	
BB		5	6	
nocall		7	8	

$$NRS = \frac{3+4+5+6}{1+2+3+4+5+6+7+8}$$

genomic variant calls (comparison)

	AA	AB	BB	nocall
AA		1	2	
AB	3	4	5	
BB	6	7	8	
nocall				

$$NRD = \frac{1+2+3+5+6+7}{1+2+3+4+5+6+7+8}$$
