# Supplementary material for: Variant discovery in targeted resequencing using whole genome amplified DNA
Source: BMC Genomics. 2013 Jul 10;14:468. doi: 10.1186/1471-2164-14-468 (PMC3716764; doi:10.1186/1471-2164-14-468)
Supplement: Additional file 4 — Figure S3. Genotype concordance matrices. Figure shows genotype concordance matrices for chr12 and and whole-exome SNP and INDEL callsets from which concordance metrics of NRS and NRD were calculated from. [file 1471-2164-14-468-S4.pdf]

Genomic (comparison)

WGA (evaluation)

	AA	AB	BB	nocall
AA	0	86	4	0
AB	48	17070	19	96
BB	0	23	11358	96
nocall	0	207	201	0

whole-exome SNPs

Genomic (comparison)

	AA	AB	BB	nocall
AA	0	91	44	0
AB	76	1030	32	40
BB	33	18	860	7
nocall	2	42	11	0

whole-exome INDELs

WGA (evaluation)

	AA	AB	BB	nocall
AA	0	0	0	0
AB	4	2623	8	6
BB	0	1	1920	11
nocall	0	6	11	0

chr12 SNPs

	AA	AB	BB	nocall
AA	0	9	6	0
AB	17	243	5	6
BB	10	5	191	0
nocall	0	9	4	0

chr12 INDELs
