# Supplementary material for: Variant discovery in targeted resequencing using whole genome amplified DNA
Source: BMC Genomics. 2013 Jul 10;14:468. doi: 10.1186/1471-2164-14-468 (PMC3716764; doi:10.1186/1471-2164-14-468)
Supplement: Additional file 6 — Figure S5. Affymetrix genotype concordance matrices whole exome. Genotype concordance matrices of WGA and genomic DNA SNP calls to Affymetrix genotypes for the whole exome capture experiment. [file 1471-2164-14-468-S6.pdf]

Affy 6.0 (comparison)

WGA (evaluation)

	AA	AB	BB	nocall
AA	7163	19	1	0
AB	17	2430	15	15453
BB	1	4	1697	9983
nocall	50	30	44	0

whole-exome capture SNPs

Affy 6.0 (comparison)

Genomic (evaluation)

	AA	AB	BB	nocall
AA	7151	29	0	0
AB	18	2440	16	15812
BB	1	5	1712	10312
nocall	62	35	36	0

whole-exome capture SNPs
