# Supplementary material for: Variant discovery in targeted resequencing using whole genome amplified DNA
Source: BMC Genomics. 2013 Jul 10;14:468. doi: 10.1186/1471-2164-14-468 (PMC3716764; doi:10.1186/1471-2164-14-468)
Supplement: Additional file 7 — Figure S6. Affymetrix genotype concordance matrices chr12. Genotype concordance matrices of WGA and genomic DNA SNP calls to Affymetrix genotypes for the chr12 capture experiment. [file 1471-2164-14-468-S7.pdf]

Affy 6.0 (comparison)

WGA (evaluation)

	AA	AB	BB	nocall
AA	565	137	0	0
AB	0	417	5	2269
BB	0	47	229	1675
nocall	19	7	3	0

chr12 capture SNPs

Affy 6.0 (comparison)

Genomic (evaluation)

	AA	AB	BB	nocall
AA	565	138	0	0
AB	0	416	4	2227
BB	0	47	231	1667
nocall	19	5	1	0

chr12 capture SNPs
